# Ribosomal Protein L23 Drives the Metastasis of Hepatocellular Carcinoma *via* Upregulating MMP9

**DOI:** 10.3389/fonc.2021.779748

**Published:** 2021-12-03

**Authors:** Minli Yang, Yujiao Zhou, Haijun Deng, Hongzhong Zhou, Shengtao Cheng, Dapeng Zhang, Xin He, Li Mai, Yao Chen, Juan Chen

**Affiliations:** ^1^ The Key Laboratory of Molecular Biology of Infectious Diseases Designated by the Chinese Ministry of Education, Chongqing Medical University, Chongqing, China; ^2^ Department of Clinical Laboratory, Shenzhen Institute of Translational Medicine, The First Affiliated Hospital of Shenzhen University, Shenzhen Second People’s Hospital, Shenzhen, China; ^3^ Department of Clinical Laboratory, The Second Affiliated Hospital, Chongqing Medical University, Chongqing, China; ^4^ Medical Examination Center, The Second Affiliated Hospital, Chongqing Medical University, Chongqing, China

**Keywords:** RPL23, HCC, metastasis, RNA stability, MMP9

## Abstract

Hepatocellular carcinoma (HCC) is one of the leading causes of cancer-related deaths globally. Tumor metastasis is one of the major causes of high mortality of HCC. Identifying underlying key factors contributing to invasion and metastasis is critical to understand the molecular mechanisms of HCC metastasis. Here, we identified RNA binding protein L23 (RPL23) as a tumor metastasis driver in HCC. RPL23 was significantly upregulated in HCC tissues compared to adjacent normal tissues, and closely related to poor clinical outcomes in HCC patients. RPL23 depletion inhibited HCC cell proliferation, migration and invasion, and distant metastasis. Mechanistically, RPL23 directly associated with 3’UTR of MMP9, therefore positively regulated MMP9 expression. In conclusion, we identified that RPL23 might play an important role in HCC metastasis in an MMP9-dependent manner and be a potential therapeutic target for HCC tumorigenesis and metastasis.

## Introduction

Hepatocellular carcinoma (HCC) is the predominant malignancy of the liver and ranks as the third most common cause of cancer-related death worldwide (1, 2). Despite the innovative progress of HCC management, most are diagnosed at advanced stage when the therapeutic options are limited. As an aggressive malignancy, HCC metastatic spread is the main obstacle to treatment and extension of long-term survival (3, 4). However, the underlying mechanisms of HCC metastasis have not yet been fully explored. Therefore, deepening the understanding of molecular mechanisms of HCC metastasis is an urgent need to develop novel therapeutic approaches.

RNA-binding proteins (RBPs) are critical regulators of gene expression by which involved in various aspects of RNA metabolism, such as splicing, modification, stability, and translation (5–8). Growing number of studies have revealed that altered RNA metabolism due to dysfunction of RBP plays an important role in cancer progression, especially cancer cell metastasis (9, 10). Human ribosomal protein L23 (RPL23), a novel RBP, has been reported that involved in various physiological and pathological processes, including cell proliferation, cell apoptosis and cycle arrest. For example, in higher-risk myelodysplastic syndrome (MDS) patients, studies showed that overexpression of RPL23 was associated with the abnormal apoptotic resistance in CD34+ cells (11, 12). Watanabe et al. demonstrated that GRWD1 expression reversed the RPL23-mediated inhibition of anchorage-independent growth in HCT116 cells by negatively regulating RPL23 *via* the ubiquitin-proteasome system (13). Meng and co-workers confirmed that RPL23-MDM2-p53 pathway could coordinate with the p19ARF-MDM2-p53 pathway against oncogenic RAS-induced tumorigenesis (14). However, much of those studies on RPL23 focus particularly on its effect in cell apoptosis, the underlying functions and mechanisms of RPL23 in HCC metastasis has been underestimated. Thus, the aim of this study is to examine the effects of RPL23 on the metastasis of HCC and to elucidate the underlying mechanisms.

Importantly, extracellular matrix degradation, mediated mainly by matrix metalloproteinase (MMP) family (15), plays a critical role in cancer cell metastasis. In particular, MMP9, a member of MMP superfamily, has been confirmed to be deeply involved in the metastasis of liver cancer (16–18). Moreover, it has also been reported that RNA binding protein HuR could increase the expression of MMP9 *via* maintaining MMP9 mRNA stability (19), indicating a potential underlying association between RBPs and MMP9 expression.

In this study, we have evaluated the expression and prognostic value of RPL23 in HCC, then further explored the effect of RPL23 in HCC migration and invasion by interacting with MMP9 both *in vitro* and *in vivo*. Further, RPL23 could enhance MMP9 expression by stabilizing MMP9 mRNA, thus to promote HCC metastasis. Taken together, our results demonstrated that RPL23 promotes HCC metastasis by regulating the mRNA stability of MMP9, implying that RPL23 could be a potential therapeutic target for HCC.

## Materials and Methods

### HCC Tissue Samples

HCC tissues and paired adjacent non-tumor tissues were obtained from 60 patients who underwent surgical resection for HCC at the First Affiliated Hospital of Chongqing Medical University in Southwest China, with the approval of the Institutional Review Board of Chongqing Medical University. The patients provided informed consent and had not received any prior radiotherapy or chemotherapy. All specimens were frozen immediately after surgery and stored in liquid nitrogen until use.

### Cell Culture

HLE and MHCC97H cell lines were obtained from the Cell Bank of the Chinese Academy of Sciences (Shanghai, China). Huh7 was obtained from the Heath Science Research Resource Bank (HSRRB). Cells were cultured in Dulbecco’s Modified Eagle’s Medium (DMEM), supplemented with 10% fetal bovine serum (Gibco-BRL), 100U/ml penicillin, and 100 μg/ml streptomycin at 37°C in 5% CO2. All the cells were examined negative for mycoplasma.

### Antibodies, Plasmids and Chemicals

The complementary DNA of full-length RPL23 was amplified by polymerase chain reaction and inserted into the *pcDNA3.1-Flag* vector at EcoRI and XhoI restriction sites. The MMP9 vector was purchased from OriGene Technologies (Rockville, MD). The specific short hairpin RNA of RPL23 (shRPL23#1:GCAAACCAGCTCAGAAATT, shRPL23#2: GAGTCATAGTGAACAATAATT) were obtained from GenePharm (Shanghai, China). Actinomycin D was purchased from Sigma (SBR00013). Mitomycin C was purchased from Selleck (S7417).

### RT-qPCR Analysis

Total RNAs were obtained from the cultured cells or tumor tissues using Trizol reagent (Invitrogen, USA). RNA was quantified by absorbance at 260 nm with a NanoDrop One (Thermoscientific). For RT-qPCR, 1 μg of RNA was reverse transcribed into cDNA using the Reverse Transcription Kit (Bio-Rad, USA). qRT-PCR was carried out using SYBR Green (Roche, Germany), and β-actin was used as control. Primer sequences used for RT-qPCR were listed in **Supplementary Table 1**.

### Western Blot Analysis

Cells were washed twice with ice-cold phosphatebuffered saline (PBS), collected in RIPA buffer with protease inhibitor cocktails (Roche, Indianapolis, IN) and lysed on ice for 15 min. Lysates were centrifuged for 5 min at 13 000 × g at 4°C and the concentration of protein was measured using a bicinchoninic acid assay (Thermo Fisher Scientific). Protein lysates were fractionated by SDS-PAGE and transferred onto PVDF membranes. The primary antibodies were as follows: rabbit anti-RPL23 (Proteintech, 16086-1-AP), rabbit anti-MMP9 (OriGene, TA326652), anti-GAPDH (Santa Cruz Biotechnology, sc-365062). Selected blots were quantified by using Image J (NIH, USA).

### *In Vitro* Assays for Migration and Invasion

Cell transwell chambers with or without matrigel (24-well plate, 8 m pores; BD Biosciences) were used to assess cell migration and invasion. For migration assay, 1 × 10^5^ HCC cells with different treatments were seeded into the upper chamber of transwells and cultured in serum-free DMEM at 37°C for 18h, while medium with 10% FBS was put into the lower chamber. For invasion assay, indicated cells were seeded in the upper chamber with matrigel-coated membrane. After a certain time, migrated or invaded cells were fixed in 95% methanol and stained with 0.1% crystal violet dye. The number of migrated or invaded cells in five different high-magnification fields (40×) were counted under an inverted microscope. To rule out effects of different cell proliferation rates that might alter the results, cells were treated with 10 ug/mL of mitomycin C for 2h before the assay was performed.

### Wound-Healing Assay

For wound-healing assay, equal numbers of HCC cells were plated into six-well plates. Until confluence, the cell monolayer was scratched with a sterile pipette tip to draw a gap, and washed twice with PBS to remove cell debris. Cells were photographed to record the wound width at 0, 24, 48 h, respectively. To rule out effects of different cell proliferation rates that might alter the results, cells were treated with 10 ug/mL of mitomycin C for 2h before the assay was performed.

### CCK8 Assay

For cell proliferation assay, CCK-8 (MedChemExpress, #HY-K0301) assay was used to assess cell viability according to manufacturer’s instructions. Different groups of cells at a density of 3000 cells/well were plated in 96-well plate with 100 μl medium and cultured for 0, 24, 36, 48, 60, 72h, then maintained in complete DMEM with 10% CCK8 reagent for 2 h at 37°C. The absorbance at 450 nm was measured by using a plate reader.

### Immunohistochemistry (IHC)

For IHC, HCC tissue samples were fixed in 10% formalin and embedded in paraffin. The tumor tissue sections were prepared, deparaffinized in xylene, rehydrated with alcohol, and then washed in phosphate‐buffered saline (PBS). In order to antigen retrieval, tissue sections were heated at 105 °C for 20 min in a citric acid buffer (0.01 M), later dealt with 3% hydrogen peroxide solution to block the endogenous peroxidase activity and blocked with bovine serum albumin (BSA) for 120 min. Next, antibodies of RPL23 and MMP9 were used to incubate at 4 °C overnight, then the tissue sections were incubated with horseradish peroxidase (HRP)-conjugated secondary antibody to detect the target protein. Antigen-antibody chromogenic reactions were developed for 12 min. After that, the slides were stained with hematoxylin and dehydration in graded alcohols and xylene. The immunohistochemical staining was analyzed by Image-Pro Plus 6.0 software.

### Luciferase Reporter Assay

The MMP9 promoter fragment was subcloned into the pGL3-basic vector to produce pGL3-MMP9-promoter. The vector was cotransfected with shCont or shRPL23. Renilla was co-transfected with reporter plasmid to normalize the transfection efficiency. After transfection for 48 h, the cells were lysed for luciferase activity measurement by using a dual luciferase reporter assay system (Promega, U.S.A.) according to the manufacturer’s instructions. The luciferase activity was determined by GloMax microplate luminometer (Promega). All the experiments were repeated at least three times.

### Nascent RNA Synthesis Assay

Cells were incubated with 0.5mM 5-ethynyl Uridine (5-EU) for 1h before harvest. Nascent RNA was captured and subjected to real-time PCR according to protocols of Click-iT^®^ Nascent RNA Capture Kit (MP10365, Thermo, MA, USA).

### Cytoskeletal Staining

The different treatment cells were seeded on the glass coverslips and incubated for 24h at 37°C. Media was removed, cells were gently washed for 3 times with PBS and fixed with 4% paraformaldehyde for 10 min at room temperature (RT). Then, cells were permeabilized with 0.5% Triton X-100 for 15 min. After washing with PBS, cells were counterstained with DyLight™ 488 Phalloidin (CST, #12935; dilution 1:40) for 10 min to stain F-actin. Subsequently, cell nuclei were stained with 4′,6-diamidino-2- phenylindole (DAPI). After air drying for 20 min, the cells were sealed with anti-fluorescent quencher. Finally, images were obtained by confocal laser-scanning microscopy using a Laser Scanning Confocal Microscopy (Leica TCS SP2).

### RNA Immunoprecipitation (RIP) Assay

RIP assay was performed using the Magna RIP RNA IP kit (17–700) from Millipore according to manufacturer’s protocol. In brief, HCC cells (2 × 10^7^) were lysed with RNA immunoprecipitation lysis buffer (Millipore, USA) and then incubated with 2 μg of rabbit polyclonal anti-RPL23 or non-immunized rabbit IgG at 4°C overnight. The RNA protein immunocomplexes were pulled down by 30ul protein A/G magnetic beads. After RNA purification, qRT-PCR was used to determine the levels of target genes.

### RNA Pull-Down Assay

RNA oligonucleotides labeled with biotin at the 5’-end were synthesized by Integrated DNA Technologies. The RNA sequences used in this study were listed as following, sense-M9-5’UTR-F: AGACACCTCT GCCCTCACC, sense-M9-5’UTR-F: GGTGAGGGCAGAGGTGTCT; sense-M9-3’UTR-F: TAATACGACTCACTATAGGG GGCTCCCGTCCTGCTTTGGC; sense-M9-3’UTR-R: TAAAGGTTAGAGAATCCAAG; M9-CDS-F: TAATACGACTCACTATAGGGATGAGCCTCTGGCAGCCCCT; M9-CDS-R: CTAGTCCTCAGGGCACTGCA. In all, 50 pmol Biotinylated RNA oligos were conjugated with 50 μl of streptavidin beads (50% slurry; Thermo Fisher) in a total volume of 300 μl of RNA-binding buffer (20mM Tris, 200mM NaCl, 6mM EDTA, 5mM sodium fluoride and 5mM β-glycerophosphate, PH 7.5) at 4°C on a rotating shaker for 2 hours. After washing three times with RNA-binding buffer, RNA-beads conjugates were incubated with 100 μg of nuclear extracts in 500 μl RNA-binding buffer on a rotating shaker overnight at 4°C.

The beads were then washed thoroughly three times with RNA-binding buffer, eluted with 30 μl 1 × SDS loading buffer and subjected to SDS-PAGE and western blot.

### Xenograft Model of Lung Metastasis

For *in vivo* tumor metastasis assays, male BALB/C nude mice (6-8 weeks old) were obtained from Shanghai SJA Laboratory Animal Co., LTD and allowed for a week adaptation upon arrival and animal experiments were conducted in the Laboratory Animal Center of Chongqing Medical University. 2 × 10^6^ MHCC97 cells infected with shRPL23 or empty vector were suspended in 40 μL of a 1:1 (v/v) mixture of a serum-free DMEM/Matrigel solution and then orthotopically implanted into the left hepatic lobe of nude mice. After 6 weeks, Mice were anesthetized with 1–3% isoflurane and killed by CO2 inhalation before killing and primary tumor volume and lung metastasis were scored. All of the procedures for handling of animals abided by the guidelines of Chongqing Medical University Animal Care Committee (reference number: 2019002).

### Statistical Analysis

The data were presented as mean ± standard deviation. All statistical analyses were conducted using GraphPad Prism8 (GraphPad) software. Unless otherwise indicated, experiments were analysed with a two-tailed Student’s t-test with a confidence interval of 95% when the number of groups equalled 2, or with a parametric ANOVA test when the number of groups was >2. The nonparametric χ2 test was used to assess the correlation between RPL23 expression and the clinicopathological parameters. The correlation between two factors were analyzed by Pearson’s test. P <0.05 was defined as statistically significant (*P<0.05; **P<0.01; ***P<0.001).

## Results

### Upregulated RPL23 Expression Is Correlated to Poor Clinical Outcomes in HCC

**Figure 1:**
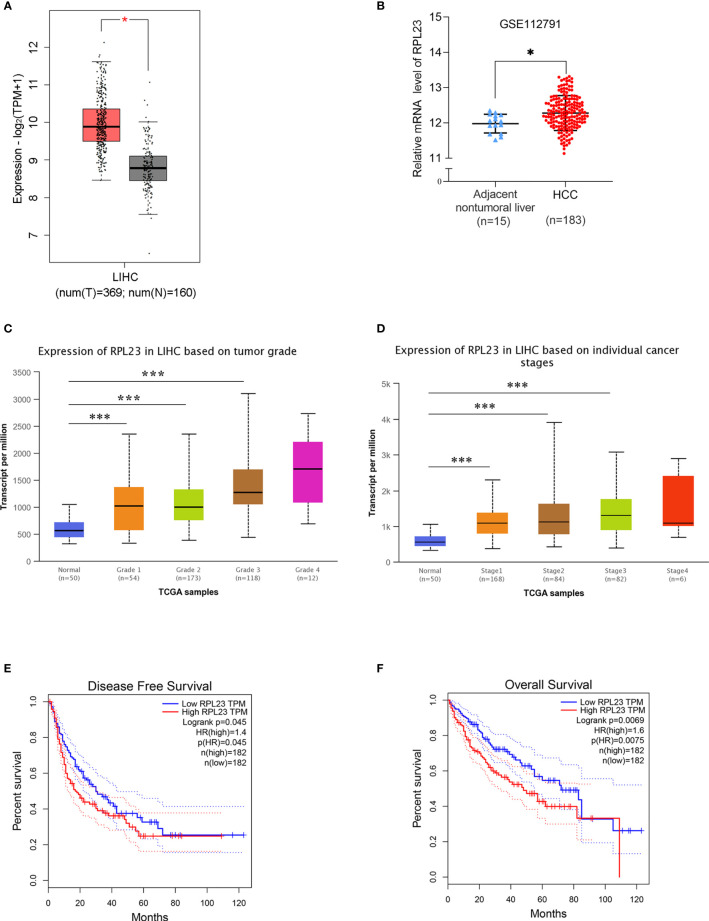
RPL23 overexpression and clinical pathological analysis in HCC tissues according to different public databases. **(A)** The mRNA level of RPL23 in the adjacent nontumor liver tissues (n=160) and primary liver tumor tissues (n=369) revealed by transcriptome sequencing of TCGA. **(B)** The mRNA level of RPL23 from RNA-sequencing data on 183 primary human HCC tissues and 15 adjacent nontumor liver tissues of GEO database in the GSE112791 cohort. **(C)** RPL23 overexpression in HCC based on tumor grade in Ualcan data-mining platform (http://ualcan.path.uab.edu/index.html). **(D)** RPL23 expression in HCC based on tumor stage. **(E)** Correlation between RPL23 expression and disease-free survival in TCGA HCC cohort. **(F)** OS curve of HCC patients based on RPL23 expression in TCGA HCC cohort. *p < 0.05, ***P < 0.001.

Based our previous RNA-sequencing data from 10 pairs of primary HCC tissues with extrahepatic metastasis (EHMH) and 10 pairs of metastasis-free HCC tissues (MFH), ribosomal protein L23 (RPL23) is extremely upregulated in HCC tissues, especially in those tissues with extrahepatic metastasis (EHMH). To broadly investigate the potential function of RPL23 in HCC, the expression of RPL23 was first analyzed in 2 published datasets, The Cancer Genome Atlas Cohort (TCGA) and Gene Expression Omnibus (GEO). As expected, RPL23 expression increased obviously in HCC tissues than that in normal tissues (**Figures 1A, B**, *p*<0.05). Moreover, RPL23 overexpression was clearly associated with advanced tumor grade (**Figure 1C**, *p*<0.0001) and late cancer stage (**Figure 1D**, *p*<0.0001). Kaplan-Meier analysis showed that patients with higher levels of RPL23 had shorter disease-free survival (DFS; **Figure 1E**, HR=1.4, *p*=0.045) and overall survival (OS; **Figure 1F**, HR=1.6, *p*=0.0069), suggesting that upregulation of RPL23 was closely related to poor clinical outcomes in HCC.

**Figure 2:**
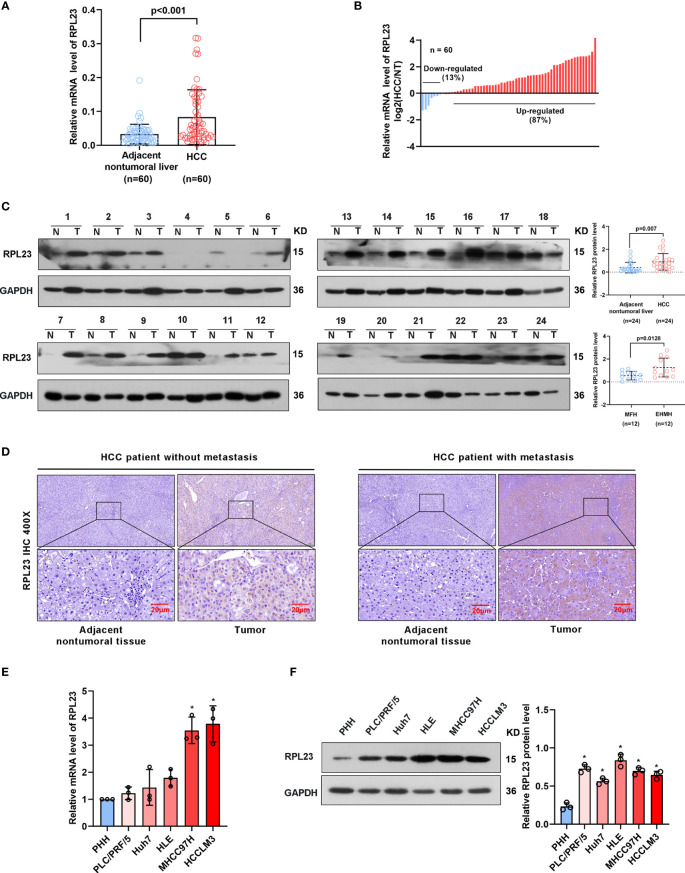
RPL23 levels increased in HCC tissues and overexpressed in different HCC cell lines. **(A)** The mRNA level of RPL23 in liver cancer was elevated in 60 paired samples of HCC tumor tissues with extrahepatic metastasis (EHMH) and metastasis-free HCC tissues (MFH), as determined by qRT-PCR analyses. **(B)** RPL23 mRNA levels in 60 HCC and paired non-tumor tissues. **(C)** RPL23 protein level in 24 paired primary HCC tissues with extrahepatic metastasis (EHMH) and metastasis-free HCC tissues (MFH) was detected by western blotting. GAPDH was used as loading control. **(D)** Representative immunohistochemical images of RPL23 staining in the paired HCC specimens with or without metastasis. (magnification, 400×) **(E, F)** qRT-PCR and Western blotting analyses of RPL23 expression in primary human hepatocytes and different HCC cell lines. GAP DH was used as loading control. Representative data are from at least three independent experiments. Data are shown as mean ± SD. *P < 0.05.

To further confirm the clinical significance of RPL23 expression in HCC, real-time PCR was performed to determine the mRNA level of RPL23 in human HCC tissues (T) and their adjacent nontumoral tissues (N) from 60 patients. The mRNA level of RPL23 was increased in human HCC tissues compared with their adjacent nontumoral tissues (**Figure 2A**), and the significant upregulation of RPL23 was observed in 87% of HCC tissues (**Figure 2B**). Consistently, the protein level of RPL23 was also increased in HCC tissue which assessed by Western blot and IHC (**Figures 2C, D**). Notably, the expression of RPL23 in EHMH was higher than that in MFH (**Figure 2D**). Moreover, the expression level of RPL23 was positively corelated to tumor vascular invasion (*p*=0.0070), lung metastasis (*p*=0.0469) and TNM stage (*p*=0.0346) in HCC (**Table 1**, **Supplementary Figure 1A**). In addition, the mRNA and protein level of RPL23 was dramatically increased in a panel of liver cancer cells compared to primary hepatocytes (PHH) (**Figures 2E, F**). Taken together, those data indicated that elevated RPL23 may involve in HCC metastasis and tumor progression.

**Table 1:** Correlation between RPL23 expression and clinicopathological characteristics in HCC patients.

Features	No. of Specimens	expression of RPL23		*p* Value
		High (n = 52)	Low (n = 8)	
**Gender**				
Males	45	39	6	0.9999
Females	15	13	2	
**Age(years)**				
≤50	24	21	3	0.9999
>50	36	31	5	
**AFP(μg/L)**				
≤20	37	33	4	0.4684
>20	23	19	4	
**Liver cirrhosis**				
Yes	38	34	4	0.4486
No	22	18	4	
**Tumor size**				
≤3cm	15	14	1	0.6657
>3cm	45	38	7	
**Multiple tumor**				
Yes	6	5	1	0.9999
No	54	47	7	
**Tumor encapsulation**				
Absent	40	34	6	0.7068
Present	20	18	2	
**PVTT**				
Yes	8	7	1	0.9999
No	52	45	7	
**Vascular invasion**				
Yes	35	34	1	0.0070*
No	25	18	7	
**Lung metastasis**				
Yes	19	19	0	0.0469*
No	41	33	8	
**TNM stage**				
I and II	17	12	5	0.0346*
III and IV	43	40	3	

*p < 0.05.

### RPL23 Silencing Inhibited HCC Cells Proliferation, Migration and Invasion *In Vitro*

**Figure 3:**
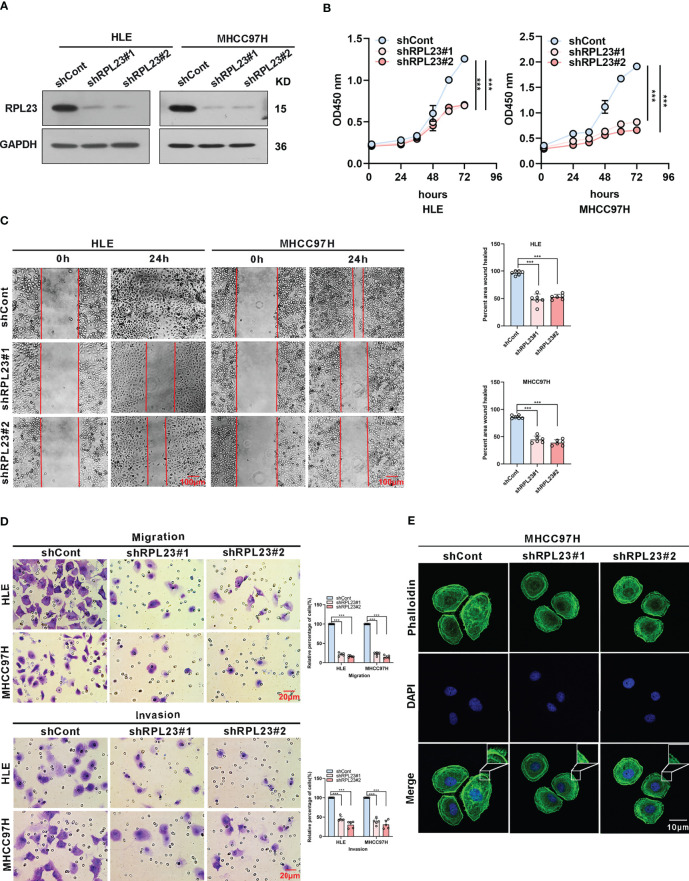
Knockdown of RPL23 significantly suppressed HCC cell proliferation, invasion and migration *in vitro*. **(A)** Western blotting was used to access RPL23 expression after transfected with negative control (shCont) or shRNA (shRPL23#1 and shRPL23#2) in HLE and MHCC97H cells. GAPDH was used as the internal quantitative control. **(B)** CCK-8 assay showed that RPL23 knockdown suppressed HCC proliferation capacity. **(C)** Wound-healing assays were performed to determine the migratory abilities of RPL23-knockdown HCC cells in HLE and MHCC97H cells. The cells were counted from 6 images. **(D)** Cell migration and invasion as measured by transwell assays were inhibited by knockdown RPL23 in in HLE and MHCC97H cells. The cells were counted from 5 images. **(E)** Phalloidin (green color) was applied for cytoskeleton staining, while DAPI (blue color) was used to mark the nuclei in RPL23-depleted MHCC97H cells. Magnification, 630×. Representative data are from at least three independent experiments. Data are shown as mean ± SD. ***P < 0.001.

To systemically evaluate the functions of RPL23 in HCC, we first examined the effect of RPL23 knockdown on HCC cell growth and invasion. We chose HLE and MHCC97H cells, which express relatively high level of RPL23 compared with other HCC cell lines. The HLE and MHCC97H cells which stably express short hairpin RNA targeting RPL23 were generated and the efficiency was confirmed by Western blot (**Figure 3A**). Compared with control cells, knockdown of RPL23 could decrease the proliferation rate in HCC cells (**Figure 3B**). Moreover, cell migration was decreased by RPL23 depletion which witnessed by wound-healing assay (**Figure 3C**). Furthermore, transwell assay also confirmed that RPL23 depletion abolished the capacity to migrate and invade in HLE and MHCC97H cells (**Figure 3D**). Those data suggested that RPL23 may participate in HCC metastasis. It has reported that cytoskeleton remodeling mediated by actin filaments plays an important role in cell metastasis (20), therefore, we examined the formation of actin filaments by using phalloidin staining. The data showed that RPL23 silencing led to the loose of actin filaments compared with the control cells (**Figure 3E**), which benefit to cell migration and invasion. Overall, these data demonstrated that knockdown of RPL23 could inhibit HCC progression by repressing cell proliferation, migration and invasion.

### RPL23 Overexpression Promoted HCC Cells Proliferation, Migration and Invasion *In Vitro*

**Figure 4:**
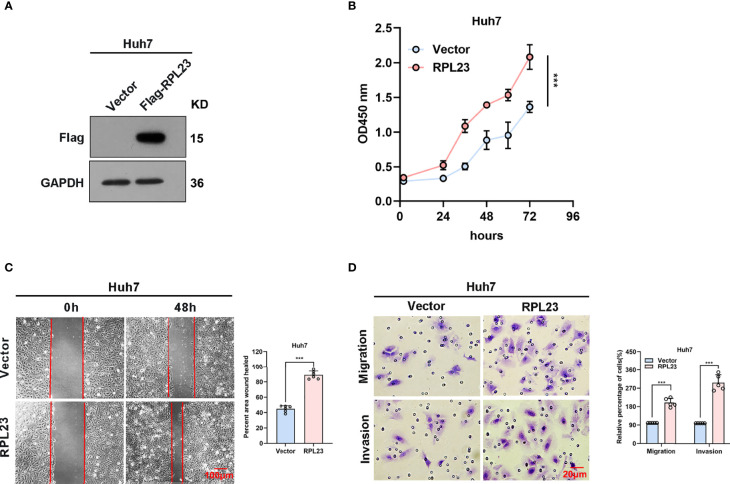
Overexpression of RPL23 promoted HCC cell proliferation, invasion and migration *in vitro*. **(A)** The overexpression efficiency of RPL23 was tested by western blotting analysis in Huh7 cells. GAPDH was used as a reference gene. **(B)** CCK-8 assay was applied to evaluate proliferation ability of Huh7 cells with expression of RPL23. **(C)** Migratory ability of overexpression RPL23 was detected by wound-healing assay in Huh7 cells. The cells were counted from 6 images. **(D)** Migratory and invasive ability were assessed by transwell assays in Huh7 cells with RPL23 overexpression. The cells were counted from 5 images. Representative data are from at least three independent experiments. Data are shown as mean ± SD. ***P < 0.001.

To further confirm the biological role of RPL23 in HCC tumorigenesis, Huh7 cells which stably express RPL23 were constructed (**Figure 4A**) and the effect of RPL23 overexpression on HCC progression were detected by series of experiments. Consistent with the findings in RPL23 depletion cells, cell proliferation rate was increased in RPL23 overexpression cells which determined by CCK-8 assay (**Figure 4B**). Next, the wound-healing assay was conducted to determine the effect of RPL23 on cell migration. As expected, overexpression of RPL23 facilitated cell migration obviously (**Figure 4C**). In addition, RPL23 overexpression cells were subjected to transwell assay and we found that ectopic expression of RPL23 promoted migration and invasion of HCC cells (**Figure 4D**). Collectively, these data indicated that overexpression of RPL23 could enhance the ability of HCC proliferation, migration and invasion.

### RPL23 Facilitates HCC Metastasis *via* Enhance MMP9 mRNA Stability

**Figure 5:**
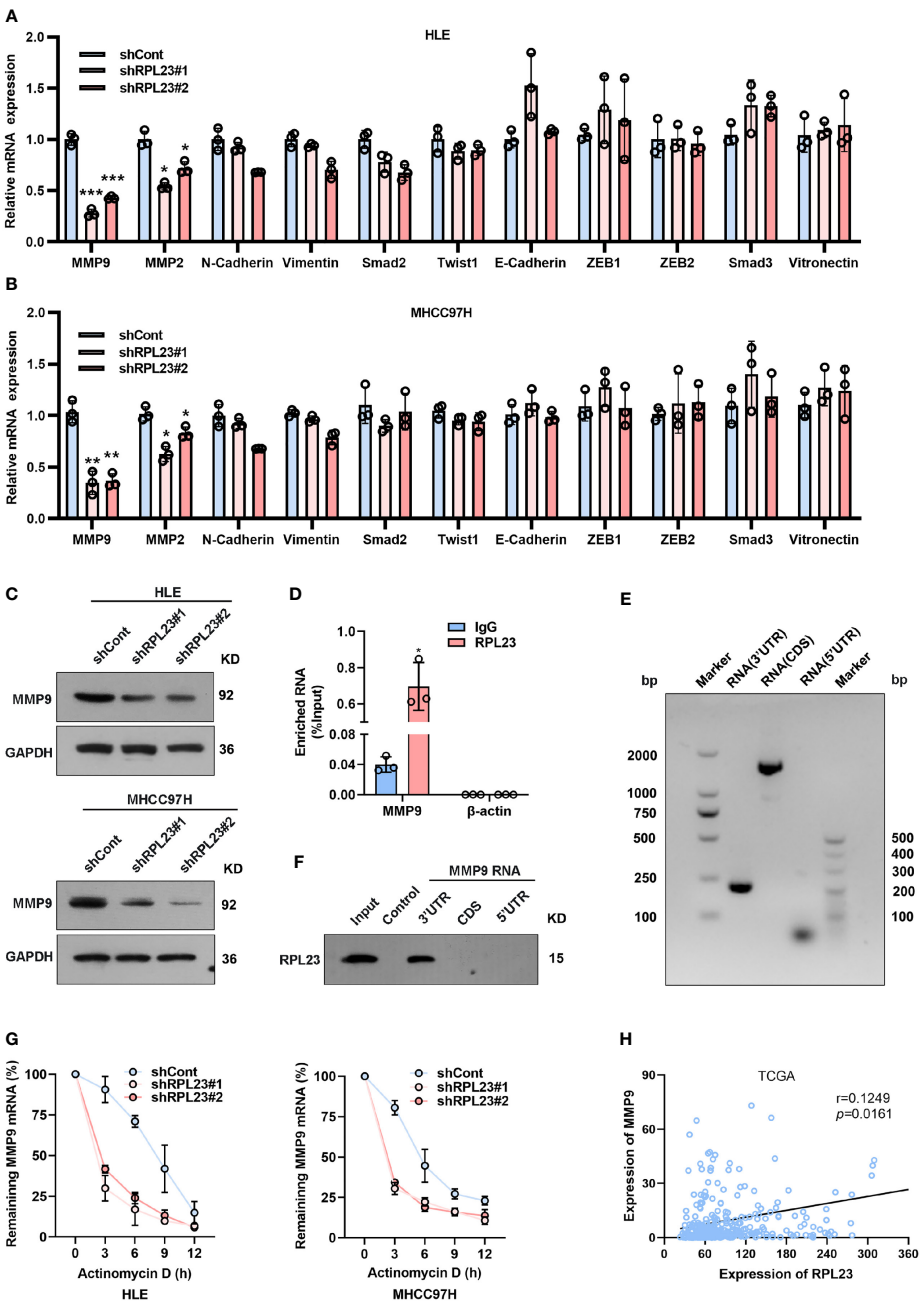
MMP9 is an essential downstream effector of RPL23. **(A, B)** EMT-related markers (MMP9, MMP2, N-cadherin, Vimentin, Smad2, Twist1 and E-cadherin) were measured on RPL23-depleted HCC cells by qRT-PCR. β-actin was used as an internal quantitative control. (***p < 0.001) **(C)** RPL23 regulated MMP9 protein expression in HLE and MHCC97H cells measured by western blot assay. GAPDH was used as a loading control for western blotting. **(D)** RIP assays showed that RPL23 directly bound to MMP9 mRNA. **(E, F)** RNA pull-down results showed that RPL23 was directly associated with the 3`UTR of MMP9 mRNA. **(E)** shows the biotinylated-MMP9-3’UTR, CDS or 5’UTR transcript *in vitro*, **(F)** shows the result of the RNA pull-down assay as analyzed by western blot. Control indicates a control pulldown containing beads only. **(G)** The half-life of MMP-9 mRNA was reduced after RPL23 knockdown in HLE and MHCC97H cells followed by treatment with 5ug/mL actinomycin D at the indicated times. Error bars represent SEM. p-values (HLE): **p = 0.00116 (shCont vs shRPL23#1), **p = 0.00296 (shCont vs shRPL23#2). p-values (MHCC97H): **p = 0.00314 (shCont vs shRPL23#1), **p = 0.00477 (shCont vs shRPL23#2). **(H)** Scatter plot between RPL23 and MMP9 mRNA level in HCC (n = 371, from TCGA database). Spearman’s correlation coefficients were calculated. Representative data are from at least three independent experiments. *p<0.05, **P < 0.01, ***P < 0.001.

Epithelial-mesenchymal transition (EMT) is a process whereby epithelial cells acquire mesenchymal features, resulting in decreased adhesion and enhanced migration or invasion. Considering that HCC cell migration and invasion could be enhanced by RPL23, we next analyzed the effect of RPL23 on EMT-associated markers in HCC cells. Based on real-time PCR data, we observed that RPL23 depletion could suppress MMP9 and MMP2 expression, while has no significant effect on other EMT-associated markers, such as N-cadherin, E-cadherin, Vimentin, Smad2 and Twist1 (**Figures 5A, B**). Due to that MMP9 is the most significant downregulated genes by RPL23 silencing, the protein level of MMP9 were further analyzed by Western blot. The results demonstrated that RPL23 silencing lead to a remarkable decrease of MMP9 protein level (**Figure 5C**). On the contrary, when RPL23 was overexpressed in Huh7 cells, expression of MMP9 was increased compared to the control cells (**Supplementary Figure 2A**), suggesting that RPL23 might regulate MMP9 expression at the transcriptional or post-transcriptional level.

To investigate the molecular mechanism underlying RPL23-regulated MMP9 expression, we first examined the effect of RPL23 on the promoter region of MMP9 by using a dual-luciferase reporter assay (**Supplementary Figure 2B**). The results revealed that RPL23 has no significant effect on MMP9 transcription. Next, considering that RPL23 belongs to the RBP family, we examined whether RPL23 could bind to MMP9 mRNA directly. RNA immunoprecipitation (RIP) assay was carried out to assess whether RPL23 directly binds to MMP9 transcripts. The results demonstrated that MMP9 mRNA was significantly enriched in RPL23-IP sample compared with IgG-IP sample (**Figure 5D**). Indeed, RNA pull-down results showed that RPL23 was directly associated with the 3’UTR of MMP9 mRNA (**Figures 5E, F**). Given that RPL23 is an RNA-binding protein which can regulate human cancer progress by influencing RNA stability (21), We then performed experiments to elucidate whether the decreased MMP9 mRNA levels were due to a change in RNA synthesis or decay. In nascent RNA capture assays, the rate of MMP9 mRNA synthesis in RPL23 silencing cells were comparable with control cells (**Supplementary Figure 2C**). Next, we treated RPL23 knockdown cells with actinomycin D to block transcription and measured decay of existing mRNAs by performing time-course real-time PCR. The results revealed that RPL23 depletion could shorten the half-life of MMP9 mRNA in RPL23 knockdown cells (t_1/2_ = 3 hours) compared to control cells (t_1/2_ = 5 hours) (**Figure 5G**). Concertedly, RPL23 overexpression could delay the degradation of MMP9 mRNA in HCC cells (**Supplementary Figure 2D**), suggesting that RPL23 regulates MMP9 mRNA stability without affecting synthesis. Moreover, RPL23 expression positively correlated with MMP9 expression in HCC based on analysis of RPL23 and MMP9 mRNA expression levels in paired tissues from TCGA (**Figure 5H**). Taken together, our data suggested that RPL23 regulate MMP9 expression *via* binding to 3’-UTR of MMP9 mRNA.

**Figure 6:**
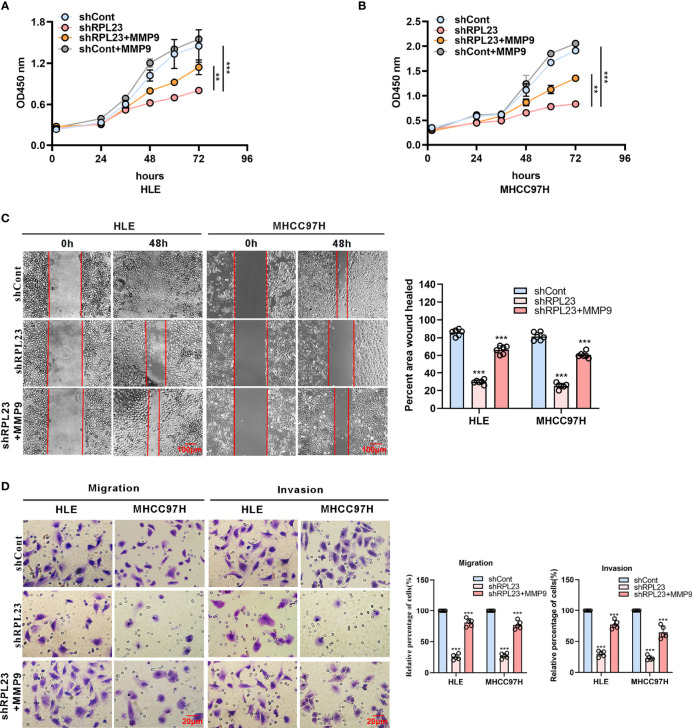
MMP9 overexpression rescues the RPL23 knockdown-induced malignant phenotypes. **(A, B)** Overexpression of MMP9 rescued the inhibition effect of decreased RPL23 on HCC cell proliferation. **(C)** overexpression of MMP-9 rescued the repression effect of knockdown RPL23 on HCC cell migration ability by wound-healing assay. The cells were counted from 6 images. **(D)** Upregulation of MMP-9 could significantly rescued the effects of decreased RPL23 in HLE and MHCC97H cells for both migration and invasion by transwell assays. The cells were counted from 5 images. Representative data are from at least three independent experiments. Data are shown as mean ± SD. **P < 0.01, ***P < 0.001.

To further study the functional role of MMP9 in RPL23-mediated HCC metastasis, we overexpressed MMP9 in RPL23 silencing cells and the effect on cell growth, migration and invasion were examined. Undoubtedly, the results showed that MMP9 overexpression markedly restored the proliferation ability of RPL23 knockdown cells (**Figures 6A, B**), and the inhibition of cell migration caused by RPL23 absence was rescued by MMP9 overexpression (**Figure 6C**). Moreover, transwell assay confirmed that the effect of RPL23 depletion on cell migration and invasion were abolished by MMP9 (**Figure 6D**). In short, we revealed that RPL23 could facilitate HCC metastasis in an MMP9 dependent manner.

### RPL23 Depletion Inhibited HCC Metastasis *In Vivo*

**Figure 7:**
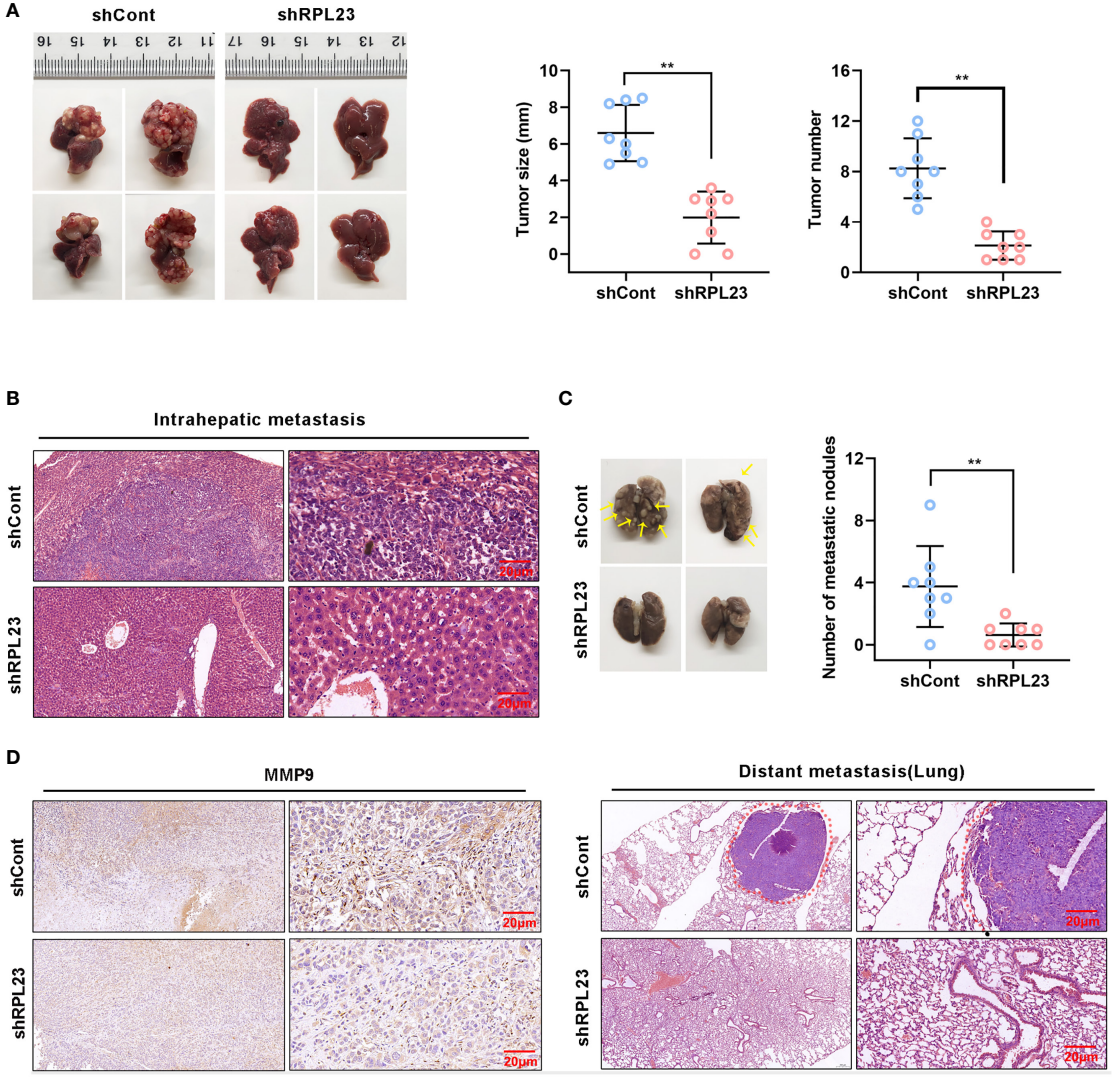
RPL23 knockdown suppressed HCC cell lung metastasis *in vivo*. The nude mice were orthotopically injected with MHCC97H cells stably depleted RPL23. **(A)** Representative images (left), volume and number (right) of xenograft liver tumor in nude mice. **(B)** The presence or absence of metastatic nodules in the liver was evaluated by Hematoxylin-Eosin staining. **(C)** Representative images of lung metastasis nodules in different groups (up), and evaluated by Hematoxylin-Eosin staining(down). **(D)** IHC assay showed MMP9 expression in metastatic xenograft model tissue in different groups. For **(A, C)**, the data were presented as mean ± SD, n = 8 in each group. **P < 0.01.

To explore the inhibitory effect of RPL23 depletion on HCC metastasis *in vivo*, MHCC97H cells which stably express short hairpin RNA targeting RPL23 were injected into the left lobe of node mouse to generate the orthotopic nude mouse HCC model. After 6-weeks injection, the mice were sacrificed and subjected to series detection. Consistent with *in vitro* observation, knockdown of RPL23 significantly decreased both the tumor growth rate and tumor size in liver (**Figures 7A, B**). Moreover, the numbers of lung metastases in RPL23-depletion mice were significantly lower compared with those of control mice (**Figure 7C**). Mechanistically, immunohistochemistry assay showed that MMP9 expression was apparently decreased in the liver of RPL23 depletion mice (**Figure 7D**). In summary, those data strongly indicated that RPL23 depletion could down-regulate the expression of MMP9, thus repressing HCC metastasis.

## Discussion

Based on an analysis of 1,225 clinical HCC samples, Dang et al. uncovered that, compared to normal adjacent liver tissues, 526 of RBPs exhibited profound differential expression and a close relationship with the poor prognosis (22). Additionally, several previous studies showed that alterations in the expression and function of RBPs in HCC could amplify the effects of cancer driver genes, accelerate tumor progression, and promote tumor metastasis (8). Up to now, about 1542 human RBPs have been experimentally validated to be involved in diverse physiological and pathological processes including cancers (5). However, the amount of well-characterized RBPs in human cancers including HCC, still remains elusive. In this study, RPL23 was identified as a tumor-promoting oncogene that plays an important role in HCC. RPL23 was significantly upregulated in metastatic HCC tissues and was positively associated with poor survival of HCC patients. RPL23 knockdown suppressed the invasive ability of HCC cells, suggesting RPL23 functions as a pro-tumorigenic factor in HCC progression.

Human ribosomal protein L23 (RPL23), a classical RBP, has been reported to be involved in a variety of human cancers including lung cancer (23), myelodysplastic syndromes (11), gastric cancer (24), and colorectal cancer (25). Additionally, some recent studies revealed that RPL23 might play an important role in cancer metastasis due to its regulatory function on RNA metabolism (16, 26). RPL23 was reported that it negatively regulated apoptosis *via* the RPL23/Miz-1/c-Myc circuit in higher-risk myelodysplastic syndrome (12), functionally inhibit the HDM2 ubiquitin ligase and thereby activate p53, leading to growth inhibition and anti-tumour effects in cases of gastric cancer (27). However, the effect of dysfunction of RPL23 on HCC metastasis still remains unclear. In the current study, besides the role of RPL23 on invasive ability in HCC cells, we found that knockdown of RPL23 resulted in the decrease of F-actin filaments, a cytoskeleton constituent that plays critical role in cancer metastasis. Meanwhile, lamellopodia could also reveal the dynamic surface extension of the cell. Furthermore, we uncovered that RPL23 silencing inhibited the distant metastasis of HCC cells *in vivo*. Taken together, our data suggested that RPL23 might play a pivotal role in HCC metastasis.

Many alterations in tumor cells are identified as contributing factors to the tumor metastasis. Among which, EMT is considered a key step driving cancer metastasis (28, 29). Our findings uncovered no significant alteration of expression of some classical EMT-related markers accompanying RPL23 depletion. However, we found MMP9, a critical MMPs protein which degrades nearly all components of ECM (30), was remarkably down-regulated after RPL23 depletion, indicating that MMP9 might be a downstream target of RPL23-mediated metastasis in HCC. Considering the classical function of binding RNA, we therefore speculated whether RPL23 regulates MMP9 *via* RNA-binding mechanisms. First, we demonstrated that RPL23 could bind to the 3’UTR of MMP9 and enhance the mRNA stability of MMP9, suggesting RPL23 regulated its target mRNA at the post-transcriptional level. Then, we found that overexpression of MMP9 markedly restored the migration and invasion abilities of RPL23 knockdown cells. We further found a significant positive correlation between RPL23 and MMP9 in HCC tissues. Here, our findings revealed a novel mechanism that RPL23 could induced HCC metastasis by stabilizing MMP9 mRNA and increasing its expression, implying a potential therapeutic approach to inhibit HCC metastasis by targeting the RPL23/MMP9 pathway. However, our findings about the interaction between RPL23 and MMP9 are still preliminary and deeper investigations need to be explored in the future.

In summary, we have identified RPL23 as a novel biomarker and prognostic factor for HCC for the first time. RPL23 associates with the 3’UTR of MMP9 mRNA and positively regulates its stability, thus leading to a pro-metastasis effect in HCC. Our finding provides a potential HCC treatment strategy that targets RPL23/MMP9 axis.

## Data Availability Statement

The original contributions presented in the study are included in the article/**Supplementary Material**. Further inquiries can be directed to the corresponding author.

## Ethics Statement

The animal study was reviewed and approved by Chongqing Medical University Animal Care Committee. Written informed consent was obtained from the individual(s) for the publication of any potentially identifiable images or data included in this article.

## Author Contributions

MY, YZ, HD, and JC designed the study. MY, YZ, HD, HZ, SC, DZ, and XH performed the experiments and analyses. LM and YC provided the materials. MY, YZ, and SC wrote the manuscript. JC critically reviewed the manuscript. JC supervised the study. All authors contributed to the article and approved the submitted version.

## Funding

This work was supported by National Natural Science Foundation of China (81861168035, 81922011 and 81871656 to JC; 31571210 to YC; 81802437 to LM), Postdoctoral Science Foundation of China (2020M683261 to STC); Creative Research Group of CQ University (CXQT19016 to JC), and Chongqing Natural Science Foundation (cstc2018jcyjAX0114 to JC).

## Conflict of Interest

The authors declare that the research was conducted in the absence of any commercial or financial relationships that could be construed as a potential conflict of interest.

## Publisher’s Note

All claims expressed in this article are solely those of the authors and do not necessarily represent those of their affiliated organizations, or those of the publisher, the editors and the reviewers. Any product that may be evaluated in this article, or claim that may be made by its manufacturer, is not guaranteed or endorsed by the publisher.
