# Corrigendum: Ribosomal protein L23 drives the metastasis of hepatocellular carcinoma *via* upregulating MMP9

**DOI:** 10.3389/fonc.2024.1373034

**Published:** 2024-03-08

**Authors:** Minli Yang, Yujiao Zhou, Haijun Deng, Hongzhong Zhou, Shengtao Cheng, Dapeng Zhang, Xin He, Li Mai, Yao Chen, Juan Chen

**Affiliations:** ^1^ The Key Laboratory of Molecular Biology of Infectious Diseases Designated by the Chinese Ministry of Education, Chongqing Medical University, Chongqing, China; ^2^ Department of Clinical Laboratory, Shenzhen Institute of Translational Medicine, The First Affiliated Hospital of Shenzhen University, Shenzhen Second People’s Hospital, Shenzhen, China; ^3^ Department of Clinical Laboratory, The Second Affiliated Hospital, Chongqing Medical University, Chongqing, China; ^4^ Medical Examination Center, The Second Affiliated Hospital, Chongqing Medical University, Chongqing, China

**Keywords:** RPL23, HCC, metastasis, RNA stability, MMP9

In the published article, there was an error in **Figure 3** as published. In **Figure 3C**, we included the wrong pictures of wounding healing assay of MHCC97H HCC cells in shRPL23#1 at 24h. We made a mistake when we dragged the original figure into the PS software, resulting in a complete repetition of images of shRPL23#1 and shRPL23#2 of MHCC97H at 24h. The representative image of MHCC97H HCC cells in shRPL23#2 at 24h shown in **Figure 3C** is accurate, and the representative image of MHCC97H HCC cells in shRPL23#1 at 24h shown in **Figure 3C** is now corrected. We re-examined the original experiment notes and confirmed that this omission did not affect the statistical results and conclusions. The corrected **Figure 3** and its caption: “Knockdown of RPL23 significantly suppressed HCC cell proliferation, invasion and migration *in vitro*” appear below.

**Figure 3:**
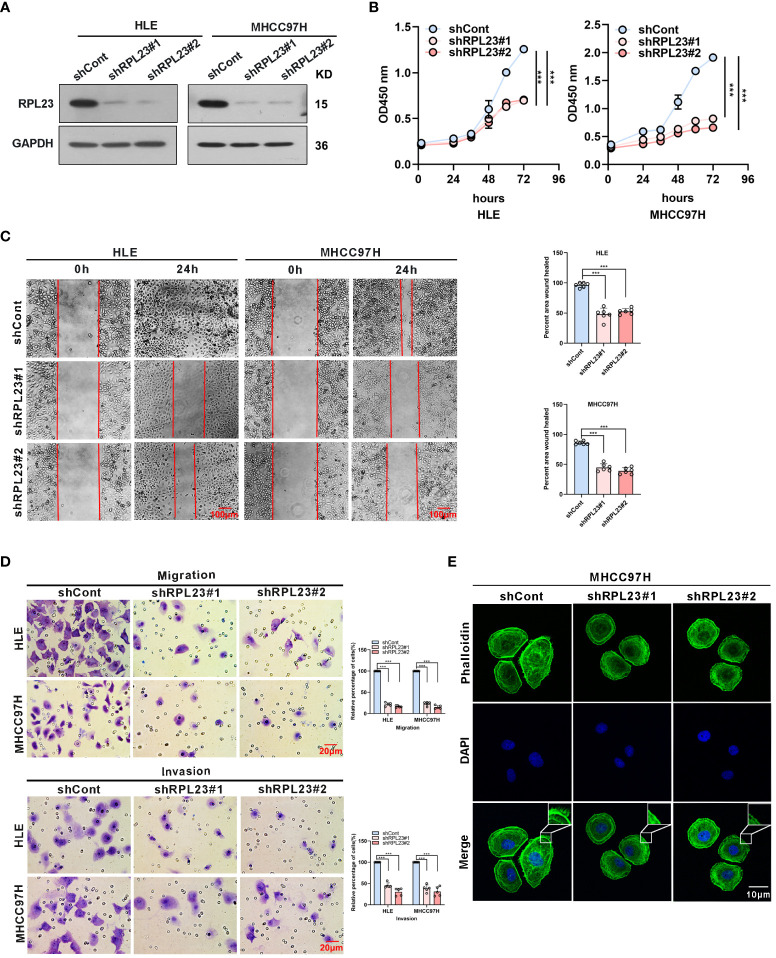
Knockdown of RPL23 significantly suppressed HCC cell proliferation, invasion and migration in vitro. **(A)** Western blot was used to access RPL23 expression after transfected with negative control (shCont) or shRNA (shRPL23#1 and shRPL23#2) in HLE and MHCC97H cells. GAPDH was used as the internal quantitative control. **(B)** CCK-8 assay showed that RPL23 knockdown suppressed HCC proliferation capacity. **(C)** Wound-healing assays were performed to determine the migratory abilities of RPL23-knockdown HCC cells in HLE and MHCC97H cells. The cells were counted from 6 images. **(D)** Cell migration and invasion as measured by transwell assays were inhibited by knockdown RPL23 in HLE and MHCC97H cells. The cells were counted from 5 images. **(E)** Phalloidin (green color) was applied for cytoskeleton staining, while DAPI (blue color) was used to mark the nuclei in RPL23-depleted MHCC97H cells. Magnification, 630×. Representative data are from at least three independent experiments. Data are shown as mean ± SD. ***P < 0.001.

There was a mistake in **Figure 3C**, an incorrect image of wounding healing assay of MHCC97H HCC cells in shRPL23#1 at 24h was used. The publisher apologizes for this mistake.

The original version of this article has been updated.

In the published article, there was a mistake in **Figure 3C**. The image of shRPL23#1 and shRPL23#2 of MHCC97H at 24h displayed were the same. The correct image of shRPL23#1 of MHCC97H at 24h appears below.

The authors apologize for this error and state that this does not change the scientific conclusions of the article in any way. The original article has been updated.

