# Supplementary material for: Ribosomal Protein L23 Drives the Metastasis of Hepatocellular Carcinoma via Upregulating MMP9
Source: Front Oncol. 2021 Dec 3;11:779748. doi: 10.3389/fonc.2021.779748 (PMC8677661; doi:10.3389/fonc.2021.779748)
Supplement: Supplementary file 1 [file DataSheet_1.docx]

**Supplementary Figure 1 The expression of VEGFR2 in HCC cells depleted with RPL23.**


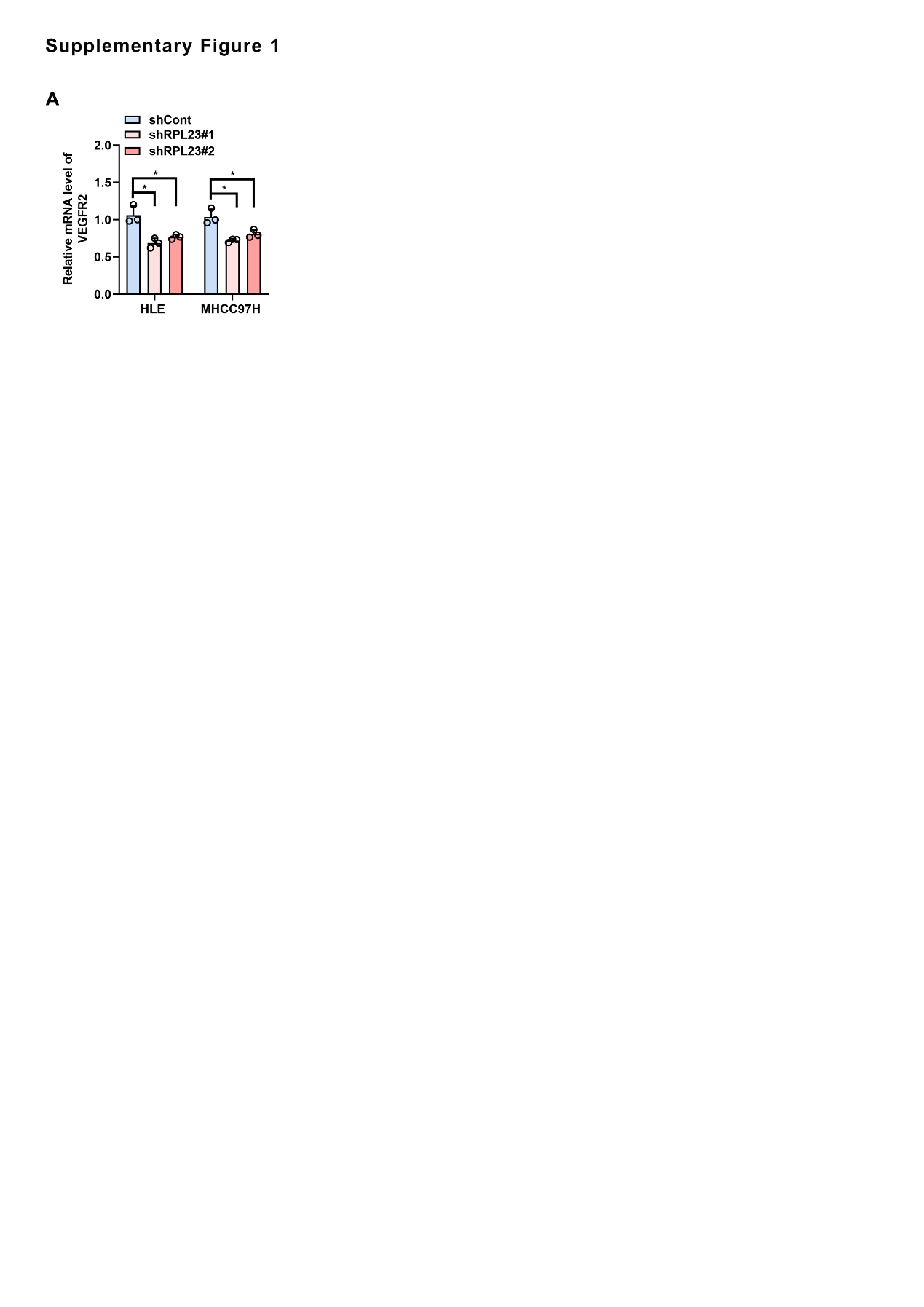


(A) The RNA level of VEGFR2 was decreased after knockdown of RPL23 in HLE and MHCC97H cells. Representative data are from at least three independent experiments. Data are shown as mean ± SD.*p<0.05.

**Supplementary Figure 2 MMP9 is an essential downstream effector of RPL23.**

**
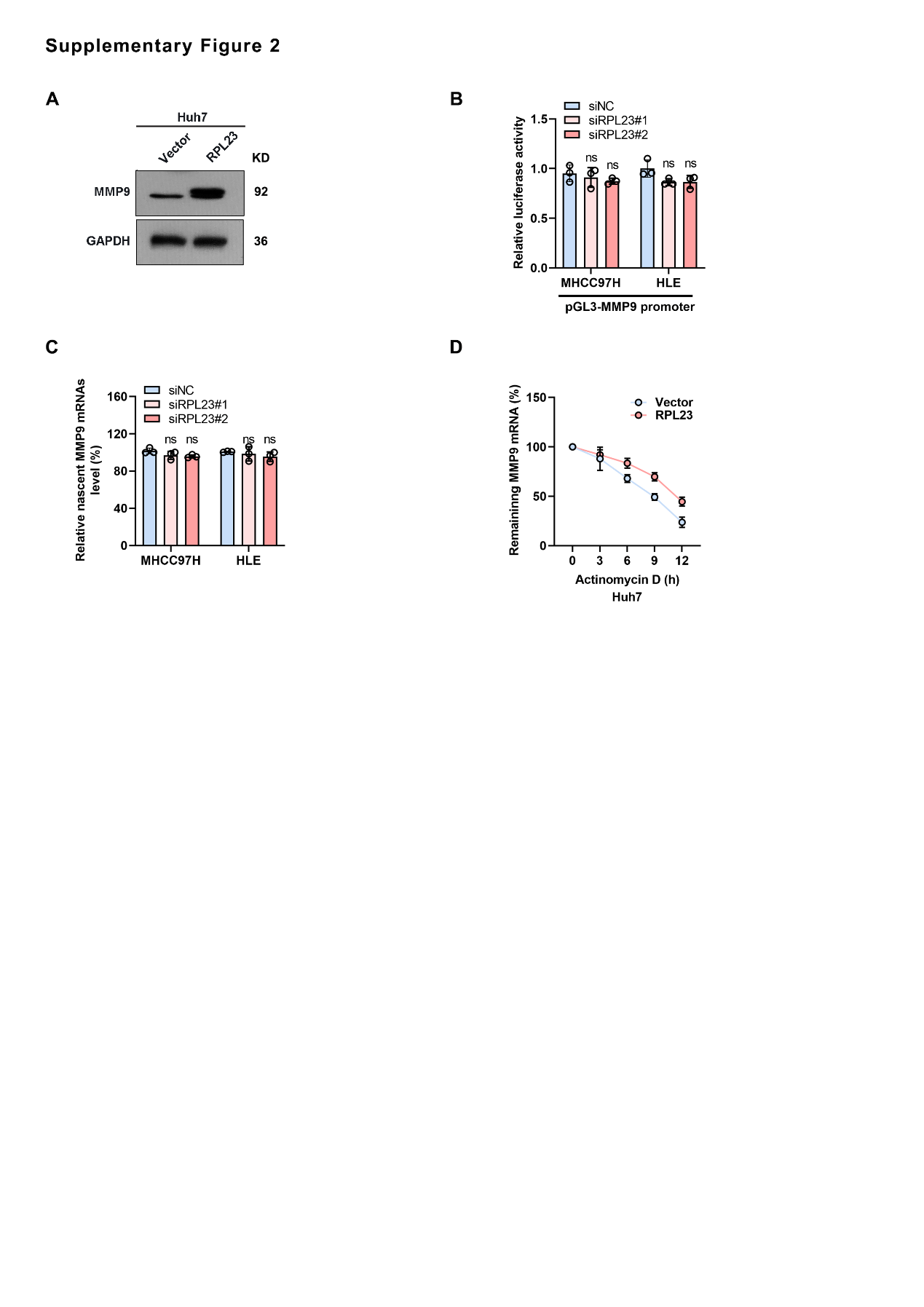
**

(A) The protein level of MMP9 increased after overexpression of RPL23 in Huh7 cells.

(B) RPL23 has no significant effect on MMP9 transcription in HCC cells using a dual-luciferase reporter assay system after co-transfection with MMP9 promoter plasmid and shCont/shRPL23 for 48 h. (C) HCC cells were incubated with 0.5mM EU for 1h after transfection with shCont/shRPL23 for 48 h. Nascent RNA was captured and subjected to real-time PCR. The rate of MMP9 mRNA synthesis in RPL23 silencing cells were comparable with control cells. (D) The half-life of MMP-9 mRNA was increased after RPL23 expression in Huh7 cells followed by treatment with 5ug/mL actinomycin D at the indicated times. **p = 0.007202 (Vector vs RPL23). Representative data are from at least three independent experiments. Data are shown as mean ± SD.**p<0.01.

**Supplementary table 1**

| **DNA oligonucleotides used for qPCR** | | | |
| --- | --- | --- | --- |
| **Gene** | **Application** | **Sense primer (5'-3')** | **Antisense primer (5'-3')** |
| *RPL23* | qPCR | AGTTTGAGAATGGAAGAATGTGGT | AGAAATGCTAACAACCTCCTACCT |
| *β-ACTIN* | qPCR | CTCTTCCAGCCTTCCTTCCT | AGCACTGTGTTGGCGTACAG |
| *MMP9* | qPCR | GCGTCTTCCCCTTCACTTTC | ATAGGGTACATGAGCGCCTC |
| *MMP2* | qPCR | TGTGTTGTCCAGAGGCAATG | ATCACTAGGCCAGCTGGTTG |
| *N-Cadherin* | qPCR | ATGGGAAATGGAAACTTGATGGC | TGGAAAGCTTCTCACGGCAT |
| *E-Cadherin* | qPCR | GCTGGACCGAGAGAGTTTCC | CAAAATCCAAGCCCGTGGTG |
| *Vimentin* | qPCR | TCCGCACATTCGAGCAAAGA | ATTCAAGTCTCAGCGGGCTC |
| *Twist1* | qPCR | CCGTGGACAGTGATTCCCAG | CCTTTCAGTGGCTGATTGGC |
| *Smad2* | qPCR | CCGCCAGTTGTGAAGAGACT | TCAGTCCCCAAATTTCAGAGCA |
| *Smad3* | qPCR | ACTGGTGCTGGGGTTAGGT | ATCCAGGGACTCAAACGTGG |
| *ZEB1* | qPCR | GCCATCATTAAAATCACTGCTTTCG | CACTGTCTGGTCTGTTGGCA |
| *ZEB2* | qPCR | AGCCTCTGTAGATGGTCCAGT | GTCACTGCGCTGAAGGTACT |
| *Vitronectin* | qPCR | CGCTTTGAGGATGGTGTCCT | GGTGCTGGAACTGGTACTCC |
